# Self-Cleaning Coatings and Surfaces of Modern Building Materials for the Removal of Some Air Pollutants

**DOI:** 10.3390/ma14092161

**Published:** 2021-04-23

**Authors:** Anna Rabajczyk, Maria Zielecka, Wojciech Klapsa, Anna Dziechciarz

**Affiliations:** Scientific and Research Centre for Fire Protection—National Research Institute, Aleja Nadwiślańska 213, 05-420 Józefów, Poland; mzielecka@cnbop.pl (M.Z.); wklapsa@cnbop.pl (W.K.); adziechciarz@cnbop.pl (A.D.)

**Keywords:** modified building materials, air pollution, self-cleaning, photocatalysis, antibacterial properties

## Abstract

Air quality is one of the most important problems of the modern world, as it determines human health and changes occurring in other elements of nature, including climate change. For this reason, actions are taken to reduce the amount of harmful substances in the air. One such action is the use of building materials with special properties achieved by the application of self-cleaning coatings and photocatalytic additives. This article presents achievements in the field of additives and modifiers for building materials, whose task is to improve air quality. Concrete, cement, paints, and facade coatings modified based on the achievements of nanotechnology have been analyzed in terms of new properties and the possibility of their application in the area of modern environmental requirements. Both positive aspects and doubts were described in the scope of the effective reduction of the amount of gases such as VOC, NO_x_, dust and microorganisms.

## 1. Introduction

Air pollution is increasing year by year. This is mainly the case in urban areas and industrial areas. Air quality is determined by the presence and concentration/amount of various gaseous substances, dusts and aerosols. Among air pollutants, the most important are CO_2_, nitrogen oxides (NO_x_), polycyclic aromatic hydrocarbons (PAHs) and volatile organic compounds (VOCs), heavy metals (including Pb, Cd, Cu, Cr, Ni, Se, Zn) and dust (PM_2.5_–PM_10_). It should be noted, however, that not only the presence of these compounds is important, but also the reactions in the atmosphere with the participation of these compounds. Organic compounds in reaction with nitrogen oxides determine ozone formation in the lower atmosphere, which is particularly important in regions with very high summer temperatures. Pollutants present in the air undergo further chemical and physical changes, creating other dangerous substances and causing deposits and patina on architectural objects, which contributes to the degradation of building materials.

One of the intensively developed ways of removing some pollutants from the air is the use of self-cleaning building materials [1]. Architectural objects made of such materials can have a significant impact not only on improving air quality but also on increasing aesthetics in urban or industrial zones.

Based on the literature data, the following types of self-cleaning building materials surfaces can be distinguished:

− Oleophobic/superhydrophobic with a contact angle >150° and reducing the amount of adsorbed soil [2,3,4];
− Hydrophilic/super hydrophilic having a contact angle <10° and facilitating the removal of water pollution, e.g., during rainfall [5];
− Oleophobic-hydrophilic with a contact angle <10° for water and an angle >150° for non-polar substances, which reduces the amount of adsorbed dirt and facilitates the removal of impurities [6,7,8];
− Photocatalytic containing additives decomposing impurities.

Obtaining such surface properties of building materials generally requires the application of appropriate coating materials or the introduction of additives that modify the composition and structure of the building material.

The purpose of this paper is to systematize information on recently developed building materials with self-cleaning surfaces, in particular biomimetic superhydrophobic or superhydrophilic surfaces as well as photocatalytic ones decomposing pollutants. A literature review was made using the following databases: Web of Knowledge, Scopus, and Google Scholar. The research also covered Espacenet, Patentscope, and Google Patents, which resulted in the presentation of selected patents relevant to the subject.

## 2. Surface Wettability

As is known, the self-cleaning properties of the surface are primarily related to the contact angle formed by the drop of liquid placed on the substrate after reaching equilibrium, which is described by the Young–Dupré equation [9]. The surface of the coating material is perfectly wetted when *θ* = 0, while it is hydrophobic when *θ* ≥ 90°. Generally, during measurements the contact angle hysteresis is determined by the difference of increasing and decreasing contact angle. The ideal state could only occur if the solid surface was completely smooth, chemically homogeneous, and the measurement was carried out slowly enough so that the state of all surfaces was as close to equilibrium as possible. In fact, roughness has a significant effect on surface wettability, as described by Wenzel and Cassie [10]. The Wenzel theory describes a rough surface completely wetted by a liquid that penetrates all substrate structures, whereas the Cassie model applies to a rough surface with a very regular structure forming the so-called composite surface in which the air is occluded in unevenness. This reduces the wettability and creates a superhydrophobic surface with a contact angle above 150° [10].

In recent years, both basic research and application works in the field of coating materials with superhydrophobic and superhydrophilic properties obtained thanks to the designed regular structure and surface chemical structure have been intensively developed [11,12]. Based on the processes and systems naturally present in the environment (e.g., lotus leaf, rice leaf, butterfly wings), a wide range of materials with designed surface properties have been designed and are still being designed. For their production, polymers, ceramics, metals or hybrid composite systems are used. An overview of such self-cleaning surfaces is presented in Table 1.

Solutions that have been used as superhydrophobic coatings on building materials will be discussed.

Lotus effect^®^ surface is one of the most known concepts of such a composite structure surface based on the lotus leaf self-cleaning behavior described by Barthlott [2,21]. The superhydrophobic properties of a lotus leaf are associated with the presence of wax particles on the leaf surface characterized by regular nano-scale roughness. Paints enabling the lotus leaf effect have found the widest application for the production of self-cleaning building materials. The superhydrophobic surface effect is usually obtained by using as a binder organic inorganic hybrid polymer materials often containing polysiloxanes or fluoropolymers and acrylic resins [22,23,24]. It is also possible to produce superhydrophobic surfaces using hydrophobic silica nanoparticles [25]. The use of rich chemistry of silicon compounds and reactive polysiloxanes allows for the use of sol-gel processes [26,27]. A superhydrophobic coating material can be fabricated by the sol–gel processing of long-chain 3-methacryloxypropyltrimethoxysilane and fluorosilanization [28]. In recent years, the use of many other methods for the preparation of superhydrophobic coatings, e.g., spin-coating [29,30], dip-coating [31,32], plasma treatment [33], chemical etching [34,35], electro-spinning [36,37] and layer-by-layer (LBL) assembly [38], has been described.

Quan et al. [39] found that in the case of superhydrophobic surfaces that are arranged obliquely, the self-cleaning process can take place in two ways, i.e., collecting particles via the water–air interface and by releasing impact droplets from the surface. In addition, they found that the wettability of the particles has no effect on the self-cleaning of superhydrophobic surfaces and a higher initial kinetic energy is needed for the droplets to remove hydrophilic dust particles [39].

It should be added that surface properties are important, as they may change under the influence of interaction with water and other external parameters. The surface is exposed to degradation and/or blockage by charges and radicals present in the air. The poor mechanical stability of some superhydrophobic surfaces determines their applicability in practice. It was found, inter alia, that the thickness of the PDMS layer is important for the photocatalytic properties of TiO_2_. The thinner the PDMS layer, the better the photocatalysis efficiency [40]. Contrastingly, Xiong [41], in his research, showed that the optimal superhydrophobic surface formed on the cathode aluminum substrate has a maximum contact angle of 161° and a maximum polarization resistance of 1591 kΩ cm^2^, indicating an excellent barrier against chemical corrosion. It was also found that the application of the electrodeposition method of thin layers of superhydrophobic cobalt stearate determines the resistance to UV radiation [41]. She et al. [42] by combining electrodeposition with chemical modification, they obtained a stable superhydrophobic surface with a cone-like structure on AZ91D magnesium alloy. The obtained surface has a very low slip angle of 1.2 ± 0.9° and is characterized by both good mechanical and chemical stability as well as good long-term durability, anti-corrosion and self-cleaning effect. The method of obtaining a superhydrophobic surface can be used on other metallic materials, including building structures [42]. Xu et al. [43] proposed another, but effective and additionally environmentally friendly method involving the use of a solution immersion method to create a superhydrophobic surface on a copper mesh. The superhydrophobic surface was obtained using the dip method. The obtained surface was characterized by a dendritic rough structure, low surface energy, while the contact angle was 155.5°. In addition, the tests carried out by the authors showed that the material has excellent: anti-fouling and self-cleaning properties, chemical stability in both acidic and alkaline solutions, and anti-corrosion effect (thanks to the air pockets that have formed between the superhydrophobic surface and the water that can block the corrosion process well) [43].

However, the use of developed materials and methods of their application or manufacture encounters considerable difficulties in relation to building materials. An important reason is that porous building materials possess very complicated surface characteristics. The water vapor permeability of treated and untreated building materials should be similar. In the event of non-compliance, the surface of the building material may be damaged and the surface layer may even delaminate. It is worth noting that in a situation where porous building materials were covered with a superhydrophobic coating, i.e., a coating with a contact angle >150°, and if there was no suitable porous structure to allow water vapor diffusion, this would not be considered an appropriate solution. At the same time, most technologies for producing or applying coatings make it impossible to use them to protect building materials. Therefore, practical applications are limited and the most widely used on the market are paints giving a superhydrophobic surface with the lotus leaf effect [44,45].

Superhydrophobic surfaces play an important role in activities aimed at protecting the facades of buildings exposed to environmental pollution. Due to their hydrophobic properties, they can be used to protect against compounds such as hydrocarbons or against the unauthorized application of substances such as paints used by the authors of graffiti on the facades. Carrascosa et al. [46] in their work combined the features of a superhydrophilic coating with hydrophobic impregnation in order to prevent water penetration into the porous structure of the substrate. The starting solution, which consisted of TiO_2_-NPs, silica oligomer (to ensure good adhesion to the substrate) and polydimethylsiloxane (as a hydrophobic agent), was sprayed onto concrete. In this way, the authors obtained a photo-induced superhydrophilic surface. Based on the conducted research, it was found that the obtained surfaces changed from superhydrophobic to superhydrophilic under the influence of sunlight. These surfaces showed underwater superolophobia, and the oil-contaminated dust was easy to clean without the use of detergents. In addition, they were characterized by adequate adhesion to the substrate and good resistance to precipitation and external exposure [46].

## 3. Photocatalysis

Building materials, in order to meet the performance requirements set in recent years, must, in addition to superhydrophobic properties, also have photocatalytic properties. Therefore, in recent years, research teams have focused on investigating building materials or paints containing photocatalytic additives decomposing impurities.

According to the definition of the International Union of Pure and Applied Chemistry (IUPAC), photocatalysis is a process in which, under the influence of UV, visible or infrared radiation, the reaction rate or its initiation changes. A necessary element is the presence of a photocatalyst, which absorbs light and is involved in the chemical transformation of reactants. Photocatalysis is classified as so-called groups of advanced oxidation processes. The semiconductor, built of valence, band gap and conductivity, is excited by absorbing radiation of a specific wavelength. The amount of energy supplied must be large enough for the electron from the valence band to be transported to the conductivity band. This process determines redox reactions. An excited electron in the conductivity band reacts with oxygen. As a result of the reaction, a superoxide anion radical is first formed, and as a result of further transformations hydrogen peroxide is formed. Ejection of the electron in the valence band creates the so-called holes (h+), which is responsible for highly hydrophilic conversion [47,48,49].

Photogenerated holes diffuse onto the surface and are trapped in places of oxygen in the network. Then, there is a reaction that leads to the formation of hydroxyl radicals. The resulting products, i.e., hydrogen peroxide and hydroxyl radicals, belong to strong oxidants, which in reaction with organic compounds can lead to the decomposition of these compounds into carbon dioxide and water. Among many materials or compounds such as ZnO, CeO_2_, SnO_2_, ZrO_2_, CdS, ZnS, WSe_2_, α-Fe_2_O_3_, SrTiO_2_, WO_3_, tested for use in photocatalysis processes, TiO_2_ is the best due to its high photocatalytic activity, physical and chemical stability in the dark [47,49,50], no corrosion, non-toxicity and availability, and low cost. Therefore, TiO_2_, known as white pigment, is most often used as an additive to building materials.

There are many crystalline forms in which TiO_2_ is present; however, tetragonal anatase and rutile are the most common [51,52]. It should be noted that not all of these forms can act as a photocatalyst. Anatase and rutile have similar titanium-oxygen bond lengths; however, the angles between oxygen-titanium-oxygen bonds are more distorted in anatase. This is a consequence of differences in the arrangement of octahedrons [TiO_6_] building individual polymorphic variants of TiO_2_. Therefore, the structure of anatase is more open than rutile, which makes the variety of anatase metastable, and has higher photocatalytic activity, while rutile is more chemically stable but less active in terms of photocatalysis. Some TiO_2_, being a mixture of anatase and rutile, have higher activity compared to pure anatase or pure rutile [53,54,55].

**Figure 1:**
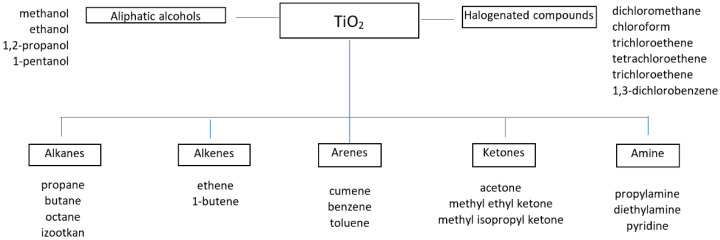
Organic pollutants removed from the air in the process of photocatalysis with TiO_2_.

The applicability of TiO_2_ is therefore determined by the crystallographic structure as well as morphology and electronic structure. Therefore, research is being carried out involving modifications of TiO_2_ to obtain materials that are efficient in terms of photocatalysis, but at the same time physically and chemically stable. TiO_2_ is used in substances for coating photocatalytic layers of sound absorbing screens placed along traffic routes, tunnel wall surfaces and concrete road surfaces, wall surfaces in operating theatres as a disinfection element. Photocatalytic layers are obtained on ceramic materials used in construction, such as bathroom tiles that allow for odor removal [56,57]. The addition of TiO_2_ allows for, among others, a reduction of various organic compounds from groups such as aliphatic alcohols, halogenated compounds, alkanes and amine (Figure 1). The decomposition of organic compounds in the photodegradation process produces less toxic products, such as CO_2_ and water. However, the effectiveness of these processes depends on the intensity of radiation, the presence of other compounds, and the presence of competing reactions [58,59].

The addition of TiO_2_ also enables the reduction of other compounds, such as NO_x_ in the air, CO_2_ photoconversion, water decomposition, photooxidation of highly toxic cyanide ions, photodegradation of a group of chemical substances that are a potential threat to the environment, which are imidazolium ionic liquids.

TiO_2_ surface chemistry is also an important issue, which affects the angle of contact of water adhering to the surface under the influence of radiation. TiO_2_-coated surfaces are ultra-hydrophilic in the course of the radiation, whereas, if the radiation stops, the surface slowly regenerates and a hydrophobic surface is created. Re-irradiation restores the ultrahydrophilic properties of the coating [60]. The adsorption of organic compounds on the surface of the TiO_2_ film affects the transition of the structure from hydrophilicity to hydrophobicity. The effective photocatalytic decomposition of these organic contaminants would allow the surface to maintain superhydrophilicity [49,61,62] and preserve the self-cleaning of the TiO_2_ film [49,58].

Also important, the contact angle with water for several metal oxides, such as WO_3_ and V_2_O_5_, decreases in response to UV radiation. Thus, the photocatalytic decomposition of pollutants adsorbed on the surface may influence the phenomenon of surface wetting [58,59,63]. One hypothesis indicates that the efficiency of photoinduced superhydrophilic conversion strongly depends on the intensity and wavelength of actinic light. The initial stage of photocalytic decomposition involves photo-excitation of electrons and generation of charge carriers [58,64]. It has also been found that the hydrophilic conversion depends on the temperature and acidity of the surface. The change of surface acidity changes the trapping efficiency of charge carriers, which in turn determines the interaction between surface hydroxyl groups and outer hydroxyl layers, and thus affects the efficiency of photo-induced hydrophilic conversion [58,59,64]. It should be added that the electronic structure near the surface is significantly different than that in the material depth, which determines many macroscopic effects and phenomena on surfaces, including surface energy (tension), adhesion forces and specific chemical reactivity of individual surfaces. This, in turn, influences interactions with other substances and the mechanisms of these interactions [65]. The activity of the photocatalyst is determined not only by the air composition and radiation intensity [66], but also by the size of the forbidden gap, and thus the structure and size of crystallites. It was found that in the case of TiO_2_ made of crystal grains with a size of 1–10 nm, the forbidden gap is greater than for a single crystal. Due to the extension of the effective forbidden gap of materials made of nanoparticles, the redox potential of photo-excited electrons and holes is higher. In addition, the shorter path of migration from the inside of such a semiconductor means that the photocatalyst is characterized by a greater number of electron–hole (e-)-(h+) pairs that are present on the surface and responsible for the efficiency of the photooxidation process. High nano-TiO_2_ activity in photocatalytic processes has been demonstrated, among others, in the processes of 1-butene gas phase oxidation or propyne hydrogenation, where n-TiO_2_ with a size of about 5 nm was used [55,62,67,68].

It should be noted, however, that titanium dioxide absorbs only ultraviolet radiation, so the photocatalytic process can only occur when exposed to such light. Therefore, the modification is carried out in such a way as to obtain properties not only increasing its durability and mechanical strength but above all the efficiency in the field of photocatalysis, e.g., by increasing the activity during exposure to visible light. It has been shown that photocatalysts that are active in visible light can be modified by introducing nitrogen, sulphur, boron, carbon or a combination of these non-metals into the TiO_2_ structure. Therefore, the TiO_2_ surface is added or modified with non-metallic admixtures, ions and metal complexes, e.g., chromium (Cr), iron (Fe), manganese (Mn), molybdenum (Mo), niobium (Nb), vanadium (V), palladium (Pd), ruthenium (Ru), doping with, for example, silver (Ag), gold (Au) and platinum (Pt) nanoparticles or mixing with other semiconductors such as CdS [50,69,70].

During the modification, three parameters are taken into account, which determine the photocatalytic efficiency, i.e., photocatalyst activity, photocatalyst carrier, i.e., the size of the surface on which the photocatalytic reaction will take place, as well as the type and power of the radiation source. The specific surface determines the number of active centres on which the adsorption process takes place, whereas, in the case of photocatalysts, the size of the specific surface depends on the amount and size of the particles of the amorphous phase and the crystalline phase being formed. It was found that with an increase in the specific surface area, the amount of OH^∙^ radicals generated, determining the speed of the process, increases significantly [50,62,69,70,71].

The new elements incorporated into TiO_2_ act as traps for photogenerated charge carriers, reducing the rate of recombination of the electron–hole pair, which may increase the efficiency of the photocatalytic process [71,72]. Photocatalysts resulting from the modification of TiO_2_ can activate TiO_2_ with visible light. In the case of impurities, integration with TiO_2_ takes place and thus modifies the composition and electronic structure. The thus obtained photocatalyst is characterized by a reduced band gap energy [71,73]. On the other hand, such substances as nanoparticles of noble metals or organic dyes absorb visible light themselves and transfer the excited electron to TiO_2_, which allows for the initiation of the photocatalysis process [71,74].

Luna et al. [71], in order to promote the photoactivity of TiO_2_ under the influence of solar radiation, modified TiO_2_ by doping with nitrogen and depositing Au nanoparticles. In addition, Au/N-TiO_2_ photocatalysts were incorporated into the silica sol to allow them to be sprayed onto three different building material substrates, i.e., limestone, granite and concrete. The authors indicated that the silica used in the work supports the adhesion of the photocatalyst to the substrate, increasing the durability of the photoactive coating, and, thanks to the porous structure, the diffusion of the involved substances to the active centers is possible. The results showed that Au-NP significantly improved the photoactivity of nanoparticles of TiO_2_, doubling the amount of NO_x_ removed in UV visible light, and the coatings increased the efficiency of removing contaminants from concrete compared to their application to limestone. However, the authors concluded that the presence of Au may cause clear color changes, which may in some way limit the application of the product to specific substrates [71].

Another example is modified TiO_2_ with carbon and nitrogen, whose sources were, respectively, methanol, ethanol and isopropanol and NH_3_ gas. The TiO_2_-N, C modified in this way was introduced into gypsum and concrete tiles and bricks in various amounts by weight, from 1% to 20% by weight. The test results showed that for materials containing 10% by weight of TiO_2_-N, C photocatalysts were the most effective in terms of photocatalysis. As a modifier, lauric acid was also used, which creates 80–90 nm thick domain structures on the surface of TiO_2_ single crystals, while irradiation with UV-Vis radiation does not change the thickness of the lauric acid layer and the surface of individual domain structures decreases. This means that the molecules located on the border of the lauric acid-TiO_2_-air contact are photodegraded. It has also been shown that photocatalysts with the highest activity contain B^3+^, S^6+^ or C-C_arom_ in their structure and have anatase structure or are amorphous, have a developed specific surface area, and their band gap width is close to or higher than, e.g., pure TiO_2_ [75,76].

An example of using n-TiO_2_ is pavement made of “anti-smog” concrete. The obtained results showed that even if the surface of the element is damaged, the concrete does not lose photocatalytic properties. Measurements carried out for two weeks showed that the NO_x_ concentration above a pavement made of ordinary concrete and concrete made of special TiOCem cement differed by an average of 49% in favor of the latter [77,78,79]. The obtained results confirm the reduction of nitrogen oxide concentrations in the air near photoactive surfaces. In a concrete product, the photocatalytically active surface under the influence of sunlight has the ability to absorb nitrogen oxides NO_x_ and their oxidation to nitrogen ions NO_3_^−^, react with calcium ions and form environmentally safe salts (such as Ca(NO_3_)_2_). It should be emphasized that such installations are effective in good sunlight; on cloudy or rainy days, or when the surface is dirty, the reduction is negligible. Efficiency also depends on location and traffic [78,79].

The thickness and depth of the layer containing the photocatalyst as well as the usable form of concrete are important for the efficiency of the catalytic process [80]. Permeable, openwork surfaces have a larger contact surface with light and air, which significantly affects the efficiency of the processes of catalytic ozone and NO_x_ removal [80]. Permeable concrete, but without gaps, means that the lack of UV-A light penetration to a depth of 50 mm eliminates the possibility of photocalysis. Without excitation of electrons by UV radiation, photocatalytic oxidation of NO_x_ cannot occur [80]. It should be noted that surface contamination significantly limits the possibility of catalytic reduction of impurities, even up to 80–90% in the case of NO_x_ reduction [62,81,82]. The pH value, cement composition, CaCO_3_ precipitation, the size and type of TiO_2_ particles added as well as the availability of water adsorbed on the surface, which plays an important role in the processes of photocatalytic NO_2_ oxidation, are also important for the efficiency of photocatalytic processes [83,84,85].

Photocatalysts based on WO_3_ are another important group of photocatalysts used in air purification processes and introduced into building materials. Among the compounds forming the volatile organic compounds (VOC) group are, among others, acetaldehyde and 2-propanol. It has been shown that it can be reduced to CO_2_ by approximately 90% after exposure to visible light for 60 min using a 110 m-Ag/WO_3_ photocatalyst [86]. However, using composite (g-C₃N₄)/WO₃ it is possible to achieve complete decomposition and removal of acetaldehyde gas from the air [87]. In the case of gaseous 2-propanol (IPA), it is possible to reduce its amount in air by using a photocatalyst being a NaBiO_3_ composite on WO_3_ with a mixing ratio of 10:100, with visible light irradiation. The use of the composite allowed for a higher degree of reduction than when only WO_3_ was used [88].

An example of other catalysts that could be used to modify building materials, because of the demonstrated properties are Fe(III)-Fe*_x_*Ti_1−*x*_O_2_ for decomposition of gaseous organic compounds, CO_2_ reduction, NO_x_ removal [89] or Ag_3_PO_4_/Cr-SrTiO_3_ to eliminate the gaseous pollutants under visible light irradiation [90].

Analysis of the available literature indicates that the basis of the catalysts used so far in building materials is primarily TiO_2_. It should be added, however, that there are many factors that can determine the efficiency of photocatalysis of a modified building material. Among others, technology for building the object (e.g., highway, sidewalks, streets, walls of buildings), imposing or introducing a photocatalytic layer, its location and thickness, and methods of producing a photocatalyst are important (Table 2).

Kamegawa et al. [97] obtained a TiO_2_ nanocomposite coating with PTFE as a result of radio frequency magnetron sputtering (RF-S) deposition. The resulting material provides excellent hydrophobicity with a photocatalytic self-cleaning effect on a fine structure substrate. This coating technique helps to suppress changes in the wettability of the surface to a hydrophilic state and, at the same time, to have sufficient photocatalytic activity for self-cleaning [97].

Among the materials used in construction, paints containing photocatalytic substances such as TiO_2_ and ZnO, which can be applied to external facades and thus improve the cleaning properties of the building, are also important. The photoactivity of ZnO paints was found to be much higher than with TiO_2_. However, during the aging process, under the influence of weather conditions, the situation changes and the paint with TiO_2_ increases its photoactivity. The polymer matrix degrades and the catalyst particles are better exposed. In the case of ZnO paints, progressive photocorrosion of ZnO particles has been observed [98]. Buildings made of photoactive concrete are known worldwide, such as the white Jubilee Church of Richard Meier in Rome, for which TX Millennium cement was used [99], air France building, Roissy-Charles de Gaulle Airport, in France [85], or Cite de la Musique et des Beaux Arts in Chambery [85,100,101]. According to Fujishima and Zhang [102], until 2003 self-cleaning tiles were used in more than 5000 buildings in Japan, including the Maru Building in Tokyo at the business centre [102].

An interesting issue is the use of material with the addition of a photocatalyst to cover road tunnels. Materials containing TiO_2_ were used in two tunnels in Europe: the Umberto I tunnel in Rome, and the Leopold II tunnel in Brussels. However, with this type of investment the problem turned out to be: lack of access to light and lack of rainfall. Photocatalytic degradation of air pollutants, especially denitrification, will only be possible if a special UV lighting system is installed. Special lighting generates additional costs and consumes energy, which reduces environmental benefits. The lack of access to water, however, causes a rapid decrease in photocatalytic activity after just a few weeks. This is because of particle deposits accumulating on the surfaces of the material that block active sites. The effectiveness of photocatalysis can be restored, partly or completely, only after washing the surface with water [103].

In the Paints and Surfaces for the Removal of Nitrogen Oxides report, the Air Quality Expert Group presented doubts about the advantages of surfaces used to remove nitrogen oxides [104]. The authors believe that it is difficult to find unambiguous and strong evidence for the effectiveness of such solutions. They claim that photocatalytic surfaces limit NO_2_ emissions only in their immediate vicinity and indicate the risk of nitric acid and formaldehyde formation at such installations. Moreover, a report from research at the Centre for Transportation Research, The University of Texas at Austin [81], indicates that although modelling results indicate the possibility of reducing pollution, field experiments did not show differences in concentration ozone between areas where modified TiO_2_ concrete was used and those where standard concrete was used [81]. It should also be taken into account that photocatalytic reactions occurring on the surface of materials and buildings may decompose and over time, and photocatalytic efficiency may change and, in the event of adverse conditions, may lead to the formation of further compounds that are dangerous to human health and the environment.

## 4. Antimicrobial Properties

From the air quality point of view, the presence of microorganisms, which not only participate in biochemical transformations but also negatively affect human health, is also important. Many microorganisms have been observed on building materials, but the most common are fungi, algae and bacteria. The presence of bacteria of the genus *Arthrobacter*, *Aeromonas*, *Brevibacterium*, *Nitrobacter*, *Nocardia*, *Bacillus, Cyanobacteria* and the classes *Betaproteobacteria* and *Gammaproteobacteria* as well as the fungi such as *Alternaria*, *Cladosporium*, *Epicoccum*, *Udeniomyces*, *Aspergillus*, *Penicillium*, *Fusarium*, *Aspergillus* has been found in building materials such as concrete, stone, glass and bricks. In contrast, *Stachybotrys* was the dominant fungus in plasterboard. The fungus *Penicillium* and yeasts with a number of other common types, such as *Aureobasidium*, *Aspergillus* spp., *Acremonium* and representatives of *Sphaeropsidales*, can be found in wood products, while metal structures can be colonized with sulfur bacteria (e.g., *Desulfovibrio* spp., *Thiobacillus* spp., *Desulfotomaculum* spp.) and ferric bacteria (*Sphaerotilus* spp. and *Gallionella* spp.). In the case of painted surfaces, bacteria of the genus *Flavobacterium Pseudomonas*, *Micrococcus*, *Streptomyces* as well as fungi of the genus *Acremonium charticola*, *A. strictum*, *A. kiliense, Acremonium* spp., *Cladosporium*, *Mycelia sterilia, Scopulariopsis brevicaulus, Verticillium lecanii, Verticillium suchlasporium, Verticillium* sp., *Fusarium* and *Penicillium* or *Aspergillus versicolor* and actinobacteria may be present [59,105,106,107,108,109].

Microorganisms on building materials, especially inside rooms, are dangerous to human health. They can produce pollutants such as allergens, spores, toxins and other metabolites that can lead to the degradation of indoor air quality and adversely affect human health, causing, among others, irritation, poisoning, superficial and systemic infections, allergies and other diseases of the respiratory system and skin [105]. A diverse population of microorganisms can also lead to the degradation of building materials, such as discoloration, cracking, corrosion, degradation of short-chain additives, material disaggregation, weakening and dissolution, and breakage of layered silicates [110].

Therefore, the addition to building materials of substances that will reduce microbial contamination plays an important role. Various inorganic substances are used to obtain concrete with antimicrobial properties, including:

− Heavy metals (Ag, Ni, W);
− Metal compounds (Ag_2_MoO_4_, CuO, ZnO, Na_2_WO_4_, NaBr);
− NORGANIX (silicate concrete sealant);
− Free HNO_2_;
− Nano-inorganic antimicrobial materials (such as Cu_2_O, CaCO_3_, TiO_2_, ZnO, CuO, Al_2_O_3_, Fe_3_O_4_) [111].

One such substance is TiO_2_, which significantly reduces the growth rate of building facades with Algae *Chlorella vulgaris*, and sometimes even completely prevents their growth. The use of TiO_2_ as a modifier of building materials also allows for an increase in the purification efficiency of algae from the *Chlorella mirabilis* and *Chroococcidiopsis fissurarum* families while flushing contaminants through rainfall. *Escherichia coli* cells on the TiO_2_ coating are destroyed after an hour of intensive exposure. It has also been found that antibacterial properties are enhanced by irradiation with low UV light and modification of TiO_2_ with copper or silver [47].

Nanoparticles of Al_2_O_3_, CuO, Fe_3_O_4_, and ZnO can also be used as cement additives, whose effects on bacteria were verified in reference to *Escherichia coli*, *Staphylococcus aureus*, *Staphylococcus aureus* (for biofilm formation), *Pseudomonas aeruginosa* and *Candida albicans*. The obtained research results indicate that, depending on the type of organism, metal oxide nanoparticles exhibit diverse antibacterial properties [112].

The test results of tests for antifungal activity against the fungus *Cladosporium* sp. on gypsum boards by Loh et al. [113] showed that ZnO particles were more effective than TiO_2_. The bactericidal properties against *E. coli* are demonstrated by the material to which SiO-TiO_2_ composite nanoparticles have been added [114].

TiO_2_ modification with nitrogen and carbon allowed for the production of concrete materials with antibacterial properties. The antibacterial activity of concrete slabs in tests with *Escherichia coli* K12 was demonstrated by slabs containing in their structure 10% by weight catalysts such as: TiO_2_/N,C_MeOH_-300, TiO_2_/N,C_EtOH_-100, TiO_2_/N,C_isoPrOH_-100 and TiO_2_/N-300 (where the carbon was from methanol MeOH, ethanol EtOH and isopropanol isoPrOH, and nitrogen was form gaseous ammonia). It should be added, however, that the antibacterial properties differ depending on the type of photocatalyst added [115].

Verdie et al. [116] conducted work on the use of a by-product of the agri-food industry as antimicrobial coatings to protect building materials against microbial proliferation. They synthesized monoglycerides (MG) from oleochemical synthons such as glycerol and fatty acids. They incorporated a specific MG molecule into a translucent aqueous coating for building materials and focused on assessing the antimicrobial potential and the target. The obtained results showed that the tested MG molecule has strong antibacterial properties against Escherichia coli CIP 53126. Verdie et al. [116] found it sufficient to apply a 1% concentration of MG to the substrate to inactivate approximately 5.6 log of *E. coli* in 24 contact hours. The use of MG as a coating limited the natural growth of microorganisms on building materials such as plasterboard.

Concrete structures used in an environment with high humidity are exposed to the influence of microorganisms through their adherence, colonization and, ultimately, destruction of the material [116].

## 5. Conclusions

Modified building materials are the answer to the worsening air quality. Thanks to photocatalysis processes, they contribute to reducing the degree of air pollution with compounds hazardous to human health, such as volatile inorganic compounds or NO_x_ or microorganisms. Combined with the self-cleaning process, the modified materials give the opportunity to extend the lifetime of the photocatalysis process and extend the life of buildings. However, it should be taken into account that the air contains not only gaseous compounds but also dusts, whose presence significantly affects the efficiency of photocatalysis and self-cleaning processes. In the case of sorption on the surface of dusts, active sites cease to fulfill their role and the reduction of pollution drops practically to zero. Therefore, the effectiveness of a given material should be verified in real conditions. Laboratory conditions do not provide the possibility of creating an air sample that reflects the composition of real air in different places (e.g., cities, industrialized areas, villages), the impact of temperature on air quality or changes in building materials that have a significant impact on the efficiency of photocatalysis and self-cleaning processes. It is therefore necessary to carry out further work on the modification of building materials in such a way that it is possible to remove contaminants at a constant level in various environmental conditions.

## Figures and Tables

**Table 1:** An overview of such self-cleaning surfaces.

Artificial Bioinspired Surface Architecture	Surface Properties	Surface Texture and Chemistry	Ref
Lotus leaf	Superhydrophobic, low adhesion	Hydrophobic epicuticular wax and papillae giving dual (micro/nano) scale roughness responsible for superhydrophobic self-cleaning effect. PDMS used for fabrication of lotus leaf replica. WCA~160° while WCA of flat PDMS surface~110°.	[13]
Gecko feet	Superhydrophobic, high adhesion	Nanopillar array with nanoscale ends (spatula) creating high adhesive forces due to van der Waals interactions. PDMS on nanopillar arrays fabricated by using electron-beam lithography covered with poly(dopamine methacrylamide-co-methoxyethyl acrylate).	[14]
Rose petals	Superhydrophobic, high adhesion	Hierarchical micro papillae and nanofolds giving sufficient surface roughness for superhydrophobic properties and high adhesive forces due to Cassie impregnating wetting state. Poly vinyl alcohol for fabrication of rose petals template. WCA~154.6°.	[15]
Fish scale	Superoleophilic in air, superoleophobic in water	The scales have oriented pappilae, 100–300 µm long and 30–40 µm in diameter, arranged in a radial direction, which provide excellent self-cleaning properties. Polyacrylamide for replication of the fish scale structrure. WCA~160° OCA < 5° in air and >~160° in water.	[16]
Shark skin	Hydrophilic, oleophilic	Small tooth-like scales. Replica of the surface by using polyvinylsiloxane WCA0° in air and 109° in water.	[17]
Rice leaf	Superhydrophobic, low adhesion, anisotropic	Dual scale (micro&nano) surface roughness arranged parallelly to surface giving anisotropic wettability. PDMS surface chemically modified with alkanethiol to enhance hydrophobicity. WCA~136°.	[18]
Snail shell	Hydrophobic, oleophilic	Surface roughness of a composite of aragonite and protein. Possibility of many applications, incl. ceramic tiles, sanitary fittings used in toilets and bathrooms and kitchens. WCA 80° OCA 10°.	[19]
Butterfly wings	Superhydrophobic, low adhesion	Micro-nano hierarchical and periodic structure. Hydrophobic fumed silica (HDK H2000) in segmented thermoplastic polydimethylsiloxane-urea copolymer. WCA~150°.	[20]

PDMS—Polydimethylsiloxane; WCA—water contact angle; OCA—oil contact angle.

**Table 2:** Examples of selected photocatalysts used in building materials to reduce pollution.

Photocatalyst	Characteristic	Application	Ref
WO_3_/SrTiO_3_/TiO_2_	at least 5 wt.% TiO_2_-NPs (in anatase form) in an amount of 0.01 to 10 wt.% in relation to the hydraulic binder	to reduction of NOx	[91]
TiO_2_/Al_2_O_3_2SiO_2_	at least 50% by weight is TiO_2_ in anatase form; the most optimal BET specific surface area in the range from 100 to 300 m^2^/g); metakaolin (Al_2_O_3_ 2SiO_2_) as a carrier—dehydroxylated form of kaolinite clay mineral obtained by calcination (specific BET surface area 12.6 m^2^/g)	to catalyze a decomposition reaction of inorganic or organic pollutants, such as: polycondensates, aldehydes, PM_10_, NO_x_ and SO_x_	[92]
TiO_2_/N,C	Mixtures of pure gypsum (Dolina Nidy Sp. Z O.O., Poland), porcelain mortar and co-modified photocatalyst TiO_2_/N, C in various amounts by weight	self-cleaning properties and photocatalyst	[93]
Ru_x_Ti_1−x_O_2_ nanobelts	RuO_2_ heteroatoms were introduced into the structure of TiO_2_ by the sol-gel method	environmental remediation—photocatalic decomposition of organic compounds	[94]
TiO_2_ (P25)	cement (Portland CEM I 52.5 R grey and white): normalized sand:water ratio of 1:3:0.5, were prepared in accordance with standard EN196-1; the Aeroxide TiO_2_ P25 (P25) catalyst was used in 5–10% wt.	NO_x_ oxidation in different conditions of humidity (RH: 0 and 65%) and illumination (Vis or Vis/UV)	[95]
TiO_2_	Portland cement (OPC) CEM I 42.5R manufactured by PJSC “Ivano-Frankivskcement” and composed of C3S: 61.35, C2S: 13.52, C3A: 6.75, C4AF: 12.02, wt.%; natural pozzolan—zeolite tuff SiO_2_, limestone powder with 95 wt.% CaCO_3_ as a micro filler; minor ultrafine additives—kaolin and TiO_2_—were added to the cementing composites; TIOXIDE^®^ R-FC5 pigment (fine crystal rutile pigment), n-TiO_2_ powder (PC-Series) with an anatase crystalline structure	improvement of mechanical properties, self-cleaning properties and photocatalyst	[96]

## Data Availability

Data sharing not applicable.
